# A Uniformity Coefficient-Based Method for Improving the Wear Resistance of Mold Ejector Pin Guide Holes via Oblique Laser Shock Peening

**DOI:** 10.3390/ma19020332

**Published:** 2026-01-14

**Authors:** Enfu Liu, Yueying Ye, Yudie Zhang, Shixu Mu, Zhilong Xu, Wenjun Jiang, Yin Li

**Affiliations:** 1 College of Marine Equipment and Mechanical Engineering, Jimei University, Xiamen 361021, Chinayeyueying@jmu.edu.cn (Y.Y.); 2 Chengyi College, Jimei University, Xiamen 361021, China; 3 China Railway First Group Urban Track Transport Engineering Co., Ltd., Xi’an 710054, China

**Keywords:** mold guide hole, oblique laser shock peening, friction performance, surface modification, compound modification

## Abstract

To address the severe wear of the hole wall and orifice in ejector pin guide holes of injection molds caused by frequent hole-shaft sliding, this study proposes a composite strengthening method that combines nitriding with oblique laser shock peening (N-OLSP). The strengthening uniformity in both circumferential and axial directions was evaluated by defining a laser shock peening uniformity coefficient (k). By strictly controlling the uniformity coefficient ratio of two adjacent spots to be no less than 0.98, the optimal step angles for circumferential and axial directions were determined. Comparative experiments were conducted on three types of samples: Untreated, Nitrided, and N-OLSP treated. The results demonstrate that N-OLSP significantly enhances both surface hardness and residual compressive stress of the guide hole, and the degree of improvement increases with a higher value of k. Among the tested samples, N-OLSP exhibited the best wear resistance at the orifice, reducing the wear rate to 0.60 μm/h. Compared with the untreated and nitrided samples, the wear rate reduction achieved by N-OLSP was 66.85% and 16.67%, respectively.

## 1. Introduction

As the key component of injection molds, the wear resistance of ejector pin guide holes directly affects the surface quality of injection-molded parts. During the ejection process, repetitive hole-shaft sliding often leads to frictional wear on the guide hole, with the orifice region being particularly vulnerable [1]. Progressive wear increases the clearance between the ejector pin and guide hole, resulting in defects such as overflow, burrs, and deformation in injection-molded parts [2,3,4]. In severe cases, wear may block the guide hole and cause ejection jamming. Therefore, improving the wear resistance of guide holes is of great importance for ensuring the quality and reliability of injection-molded products.

To enhance the wear resistance of guide holes, several engineering approaches have been adopted, including the use of high-hardness substrate materials [5], embedding self-lubricating bushings [6,7], heat treatment [8,9], and cold extrusion processing [10,11]. Although high-hardness material substrates (e.g., powder metallurgy steel) [5] can significantly improve the resistance to plastic deformation, they suffer from drawbacks such as high processing difficulty, distortion during heat treatment, brittle spalling, and increased cost. Studies on embedded bushings [6,7] have revealed that unstable frictional torque during shaft movement reduces fit accuracy, and bushings are also prone to loosening. Su and Wang [8,9] found that although gas carburizing can improve the hardness of small hole, the limited gas exchange within small-diameter holes leads to uneven carbon potential, resulting in a non-uniform hardened layer and limited wear resistance. In addition, Ge and Gong [10,11] showed that the cold extrusion process can increase residual compressive stress on the inner wall of hole, thereby inhibiting microcrack propagation during wear. However, due to the limited cold extrusion plastic deformation of the small hole, its contribution to improving the wear resistance of the small hole is very limited. These findings suggest that while various strengthening methods are effective for large-diameter holes, their applicability and performance enhancement in small-diameter guide holes require further investigation.

Laser shock peening (LSP) strengthens materials by bombarding surfaces with plasma shock waves generated by high-power pulsed lasers, inducing surface plastic deformation that enhances hardness and wear resistance [12,13,14]. Li et al. [15] reported a 39.5% reduction in wear rate after optimizing laser parameters to form a dense surface structure. Wang et al. [16] found that LSP reduced surface spalling as well as the depth and width of wear furrows. To further improve surface wear resistance, composite strengthening approaches combining heat treatment with LSP have been investigated [17,18]. Ren and Wang [17,18] demonstrated that high-density dislocations are formed on the material surface after nitriding combined with LSP, which leads to significant improvements in wear resistance. However, when LSP is applied to inner holes, the phenomenon of oblique laser shock peening (OLSP) occurs, and its strengthening effect has not been thoroughly studied.

The effect of laser incident angles on strengthening performance has been examined in several studies. Zeng et al. [19] investigated laser incident angles ranging from 30° to 75° and found that larger angles reduce laser power density, leading to weaker strengthening effects on plates. Tang et al. [20] applied OLSP to engine blades and observed significant fluctuations in residual compressive stress when the laser was not perpendicular to curved surfaces. These results indicate that deviations in laser incident angles on inner holes or irregular curved surfaces markedly affect the uniformity of residual compressive stress distribution, and thus must be carefully controlled.

However, current research on laser shock peening of inner holes is predominantly focused on medium and large holes, with relatively little attention given to small holes such as guide holes in injection molds. To address this gap, this study aims to investigate the strengthening effect of a combined N-OLSP treatment on such small guide holes. Based on the operational method of laser shock peening for inner holes, uniformity coefficients (k) for circumferential and axial directions are proposed. An increase in either the circumferential curvature or the axial depth of a small hole reduces the uniformity coefficient, thereby decreasing the strengthening effect. By strictly controlling the step angles of laser incidence, the uniformity of the peening effect for circumferential and axial directions can be ensured. The influence of axial uniformity coefficient on N-OLSP performance is evaluated using hardness distribution, residual compressive stress field, metallographic microstructure, and wear rate as key performance indicators.

## 2. Experimental Materials and Methods

### 2.1. Experimental Materials

**Figure 1:**
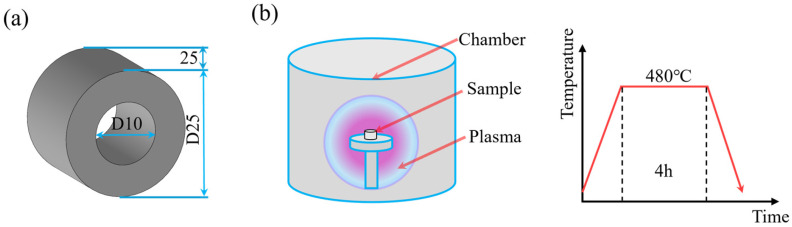
Schematic diagram of (**a**) specimen geometry; (**b**) heat treatment process.

The specimen geometry was designed as a bushing with an inner diameter of 10 mm, an outer diameter of 25 mm, and a length of 25 mm, as shown in Figure 1a. H13 hot-work die steel was selected as the specimen material for this study. The H13 steel exhibits excellent thermal stability and wear resistance, making it suitable for injection molding, die casting, extrusion, and other tooling applications. Its chemical composition is listed in Table 1. As seen from Table 1, the carbon content of H13 steel is 0.46%, which belongs to medium carbon steel. Therefore, the nitriding method is selected for the specimens.

### 2.2. Nitriding Heat Treatment

According to the standard heat treatment process for H13 steel, the specimens were first quenched at 1050 °C and then tempered at 500 °C. To further improve the hardness of the inner hole, vacuum ion nitriding was performed using a dedicated nitriding furnace. During the process, nitriding gas was introduced with a flow ratio of N_2_:H_2_ = 450 mL/min:150 mL/min under a pressure of 290 Pa. The specimens were heated to 480 °C, held for 8 h, and finally cooled along with the furnace, as show in Figure 1b.

### 2.3. Oblique Laser Shock Peening

When a laser irradiates a curved surface, only the component of the laser beam normal to the surface contributes to the shock peening effect. Therefore, when a single laser beam with uniform energy density is applied to a curved surface, the normal components vary at different points, leading to non-uniform shock peening within the irradiated spot. To quantify this effect, the uniformity coefficient (k) is defined as the ratio of the normal component of the laser beam at a given point to the maximum normal component within the same strengthened area. Therefore, a value of k closer to one corresponds to a more perpendicular laser incidence on the inner hole surface, thereby ensuring a more effective strengthening performance.

#### 2.3.1. Analysis of Circumferential Uniformity of Laser Shock Peening for Guide Hole

**Figure 2:**
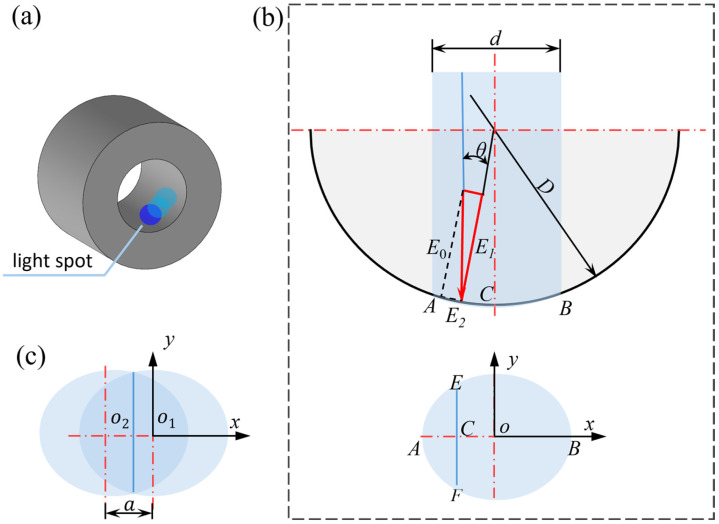
Schematic diagram of vertical laser shock on guide hole: (**a**) schematic diagram of circumferential spot; (**b**) schematic diagram of circumferential decomposition of a single laser beam; (**c**) expanded view of a single spot on the circumference.

To analyze the circumferential strengthening uniformity of the guide hole curved surface after LSP, a single laser beam was vertically irradiated onto the guide hole surface. As shown in Figure 2a, the arc AB represents the LSP region. Figure 2b presents the corresponding expanded view of the elliptical spot, where the normal laser shock energy along on the *y*-axis reaches its maximum. According to the definition of k, the circumferential uniformity coefficients of the elliptical spot along the line segment EF (where $x=x_{c}$) can be expressed as

(1) $$k_{x}=\frac{E_{1}}{E_{0}}=\cos\theta$$

where $E_{1}$ denotes the normal component of the laser beam at $x=x_{c}$ within the elliptical spot, $E_{0}$ is the maximum normal component at x=0, and θ is the angle between the incident laser beam at $x=x_{c}$ and the local surface normal.

From Equation (1), it follows that as θ increases, $k_{x}$ gradually decreases. Thus, the circumferential uniformity coefficients at points A and B are the smallest, while the circumferential uniformity coefficient at the short semi-axis ($k_{x}=1$) is the largest. While a higher $k_{x}$ improves peening effectiveness, a value exceeding one is unfavorable as it necessitates excessive spot overlap. This leads to localized energy concentration, thereby risking damage to the ablative layer, material overheating, and a loss of the guide hole’s fitting precision. Therefore, to balance peening efficiency with process safety, $k_{x}=0.98$ was selected as the target threshold for effectiveness while avoiding detrimental over-peening.

According to Equation (1), when two elliptical spots (as illustrated in Figure 2c) overlap at $x=x_{D}$, the uniformity condition is satisfied if the angle θ between the laser beam and the surface normal of the inner hole is less than 10°. Under this condition, the center distance a between two elliptical spots is less than 1.74 mm, and the circumferential step angle γ is less than 20°. By rotating the bushing at this step angle during OLSP, $k_{x}$ can be maintained above 0.98.

#### 2.3.2. Analysis of Axial Uniformity of Laser Shock Peening for Guide Hole

**Figure 3:**
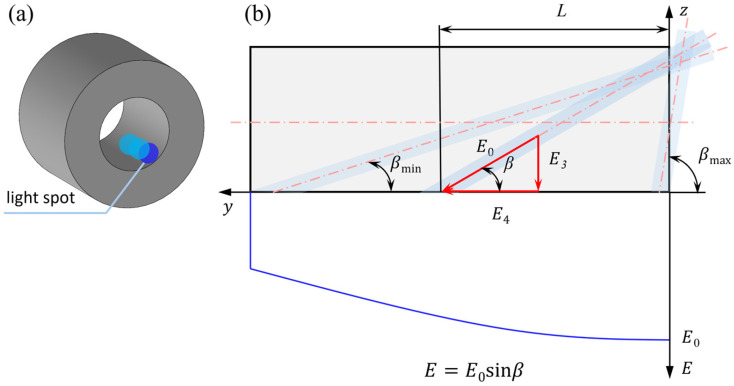
Schematic diagram of oblique laser shock on guide hole: (**a**) schematic diagram of axial spot; (**b**) axial decomposition diagram of OLSP for the guide hole.

Due to the enclosed structure of the guide hole, the laser can only be incident at an oblique angle to strengthen its inner surface, as shown in Figure 3, and the axial uniformity coefficient is expressed as

(2) $$k_{y}=\frac{E_{3}}{E_{0}}=\sin\beta$$

where $E_{3}$ denotes the normal component of laser beam at y=L within the elliptical spot, and β is the deflection angle of the laser beam relative to the guide hole axis at y=L. From Equation (2), it can be seen that as the laser deflection angle β decreases, the laser strengthening region extends deeper along the axial direction, while the normal component of the beam decreases. Consequently, the axial uniformity coefficient $k_{y}$ also decreases, as illustrated in Figure 3. The relationship between the axial strengthening depth L of the guide hole and the laser deflection angle can be expressed as

(3) $$L=D\cot\beta -\frac{d}{2\sin\beta }$$

where *L* is the axial strengthening depth (i.e., the distance from the center of the obliquely incident laser beam at angle β to the orifice, in mm), D is the diameter of the guide hole (mm), and *d* is the diameter of the laser beam (mm).

After completing one full circumferential OLSP cycle, the bushing and laser are relatively shifted by one axial step angle to strengthen the next annular surface. As the axial deflection angle of laser increases, the axial uniformity coefficient gradually decreases. For axial peening, the inevitable decrease in incidence angle with depth prevents $k_{y}$ from reaching the 0.98 threshold at all points. Instead of pursuing a constant high value, we focused on ensuring a uniform gradient of decay. To ensure axial uniformity, the axial step deflection angle between adjacent laser beams must be strictly controlled so that the ratio of the axial uniformity coefficients of two adjacent circles remains no less than 0.98. This approach guarantees a consistent and controlled decrease in peening intensity along the depth, maintaining both process uniformity and overall strengthening efficiency.

For this purpose, the deflection angle β of the first laser beam was set to 85°, and its axial step angle ${\Delta \beta }_{2}$ to 5°. At this time, the axial uniformity coefficient was calculated as $k_{y}=0.996$. After peening the circumferential surface near the orifice, the second laser beam was deflected by ${\Delta \beta }_{2}$ to cover the adjacent axial region. To satisfy the axial uniformity condition for subsequent layers, the step angle ${\Delta \beta }_{n}$ of the *n*th laser beam (n≥2) must satisfy the following equation:

(4) $$\Delta \beta_{n}\;=\;\mathrm{arc}\sin(0.98^{n-1})\;-\;\mathrm{arc}\sin(0.98^{n})$$

From Equations (2)–(4), it can be deduced that as the number of laser beams n increases, the axial deflection angle becomes larger, the axial peening depth *L* increases, and the axial uniformity coefficient decreases. When the axial uniformity coefficient falls to $k_{y}\leq 0.5$ (corresponding to β=30°, L=15.8 mm), the peening effect becomes negligible, and further peening of the inner hole is unnecessary.

#### 2.3.3. Oblique Laser Shock Peening Experiments

**Figure 4:**
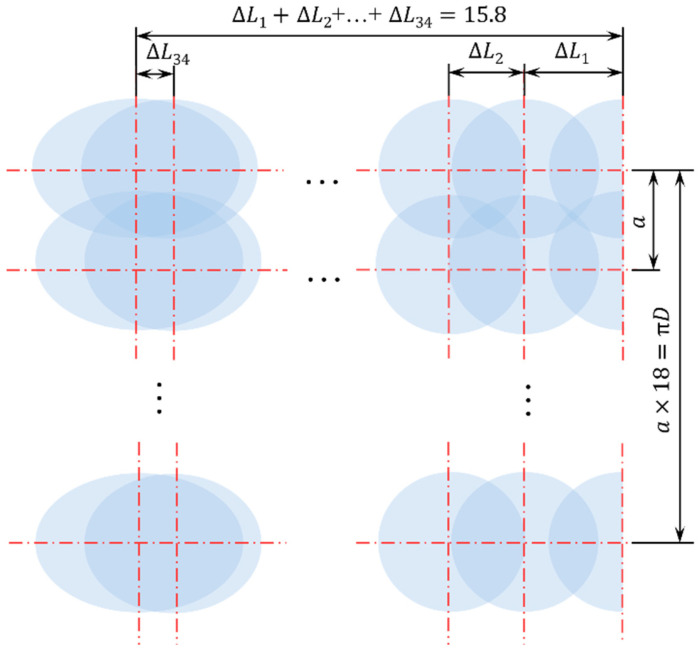
Developed view of laser array in effective peening region of inner hole.

Following both circumferential and axial OLSP, the inner cylindrical surface was effectively peened. Figure 4 shows the peened region and the developed view of the laser shock array.

**Figure 5:**
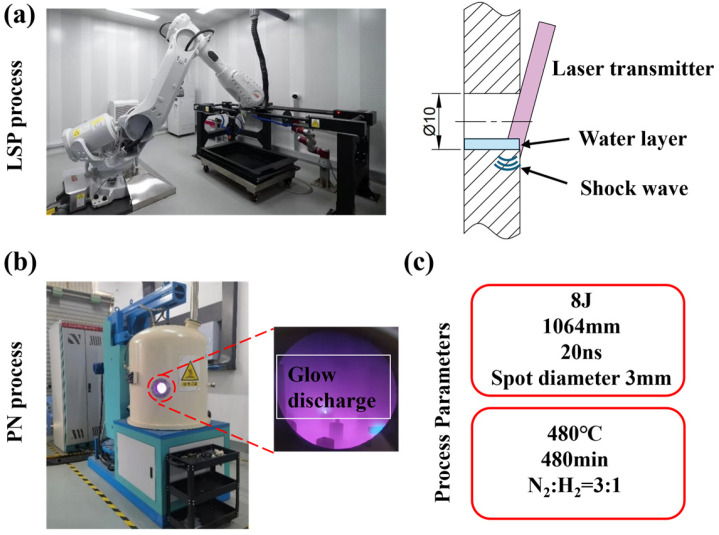
Experimental process: (**a**) schematic diagram of the laser shock peening (LSP) experimental setup and principle; (**b**) ion nitriding experimental setup; (**c**) experimental parameters.

Based on the above analysis, the OLSP experiment was conducted on the inner hole of the bushing after nitriding treatment, and parameters are listed in Table 2. During the LSP treatment, a water layer was used as the confining layer, while black glue was employed as the absorption layer. The entire experimental procedure is shown in Figure 5.

### 2.4. Testing Methods

#### 2.4.1. Microhardness

**Figure 6:**
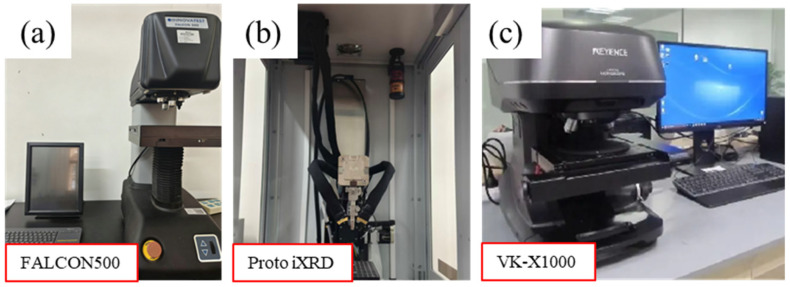
Test equipment for measurement of (**a**) microhardness; (**b**) residual stress and (**c**) metallographic.

Microhardness was measured using an FALCON500 Vickers hardness tester (INNOVATEST Shanghai Co., Shanghai, China), as shown in Figure 6a. A test load of 0.1 kg-f/mm^2^ and a dwell time of 10 s were applied. The first measurement was taken at the entrance of the guide hole. Then, additional measurements were taken along its axis at 1 mm intervals. At each point, three repeated measurements were performed, and the average value was recorded as the final hardness.

#### 2.4.2. Residual Stress

Residual stress was characterized using a Proto iXRD high-speed residual stress analyzer (Proto, LaSalle, ON, Canada) with Cr-kα radiation source, V filter, 3 mm collimator diameter, {211} diffraction plane, 1 s exposure time, 10 exposure times, and 2θ diffraction angle range of 137–144°, as shown in Figure 6b. Saturated NaCl solution was used for electropolishing to remove the material layer by layer during the test, and the final experimental data were also averaged from three measurements.

#### 2.4.3. Metallographic Observation

The cross-section sample was first mechanically polished using silicon carbide sandpaper (3M China Co., Guangzhou, China), progressively from 120 to 1200 grit. Then, it was finely polished on a cloth with diamond spray suspension. Subsequently, the sample was etched with a 4% nitric acid solution at room temperature for 5 s. Finally, the surface microstructure was observed using a VK-X1000 metallographic analysis system (Keyence, Osaka, Japan), as shown in Figure 6c.

#### 2.4.4. Wear Tests

**Figure 7:**
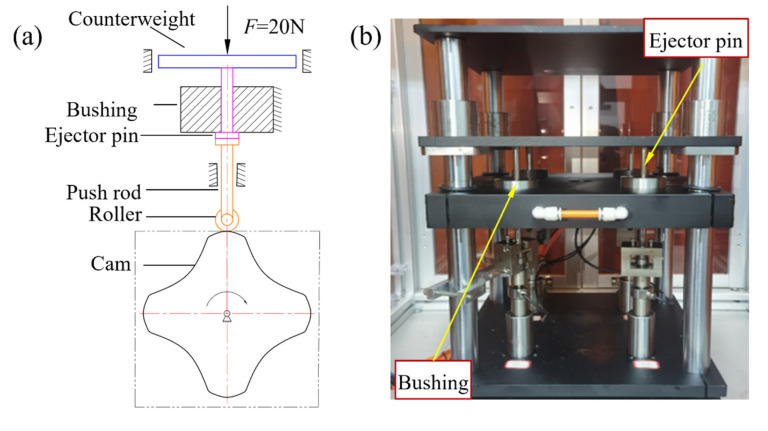
Hole-shaft friction and wear testing machine: (**a**) schematic diagram; (**b**) physical diagram.

To simulate the actual service condition of the guide hole, a cam mechanism was employed to replicate the demolding process of injection-molded parts. The working principle is shown in Figure 7a. The motor drives the cam to rotate at a specified angular velocity, which in turn drives the roller push rod to actuate the ejector pin in a cyclic, reciprocating up-and-down motion, simulating the ejection of the molded part. A force of 20 N was applied to the ejector pin through counterweight block to replicate the demolding force during the ejection process.

The ejector pin shaft specimen was made of H13 steel, with a surface hardness of 536 HV0.1. The friction and wear experiments were conducted on three specimen groups: Untreated, Nitriding, and Nitriding-Laser, with three parallel specimens set for each group. To ensure a valid comparison of wear resistance, all tribological pairs were prepared with identical geometric surface conditions (Ra < 0.2 μm) and dimensional tolerances (H7/g6) prior to surface treatment. To evaluate the effect of different treatment processes on the wear resistance of the bushing inner hole, a 70 h (approximately 1 million cycles) hole-shaft friction and wear test was conducted in this study. To improve experimental efficiency, the testing machine was capable of performing simultaneous wear tests on four different specimens. The experimental setup is shown in Figure 7b.

## 3. Results and Discussion

To evaluate the effect of N-OLSP on the wear resistance of the bushing inner hole, three groups of specimens were prepared. The first group, denoted Untreated, was not subjected to any peening process. The second group, denoted Nitriding, underwent only nitriding treatment. The third group, denoted Nitriding-Laser, was treated with both nitriding and N-OLSP. The mechanical properties of the guide holes in these three groups were systematically compared and analyzed.

### 3.1. Analysis of Axial Uniformity Coefficient and Hardness

**Figure 8:**
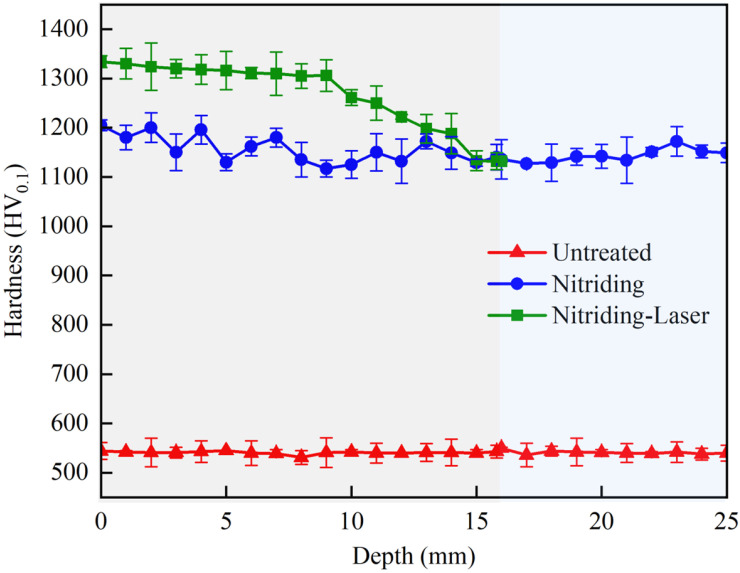
Axial distribution of inner hole surface hardness.

Hardness is one of the key indicators for evaluating the wear resistance of components [21,22]. To investigate the influence of k on the mechanical properties of the inner hole, the axial hardness of three specimen groups was measured. As shown in Figure 8, the maximum hardness for the Untreated specimen was approximately 544 HV_0.1_. After nitriding treatment, the hardness increased to 1205 HV_0.1_, representing a 121.5% improvement. This significant enhancement can be attributed to the precipitation of Fe_3_N nitrides within the inner hole [23,24,25], as well as the combination of nitrogen with chromium in H13 steel to form Cr–N compounds [26]. Together, these effects result in solid solution strengthening [27,28], thereby substantially improving the hardness. For the Nitriding-Laser specimen, N-OLSP further increased the hardness, with the highest value observed at the orifice (L=0), reaching 1334 HV_0.1_, with an 11.2% increase compared to the Nitriding specimen. Compared with the relatively stable hardness profile of the Nitriding specimen, the N-OLSP specimen exhibited hardness that was strongly correlated with the axial uniformity coefficient. Near the orifice region, where the peening uniformity coefficient is the highest, the hardness significantly exceeded that of the Nitriding specimen. As the peening depth L increased (corresponding to a gradual decrease in the axial uniformity coefficient), the hardness of the N-OLSP specimen decreased and eventually approached the hardness level of the Nitriding specimen.

### 3.2. Axial Distribution of Residual Compressive Stress

**Figure 9:**
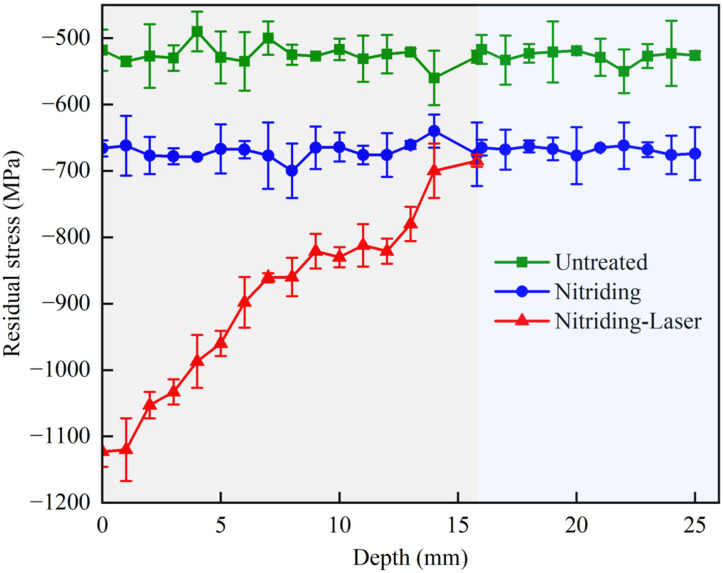
Axial residual stress distribution of specimens.

Surface residual compressive stress can inhibit crack propagation during wear and reduce material spalling, thereby improving the wear resistance of materials [29]. To investigate the influence of k on the mechanical properties of the inner hole, the residual compressive stress of three specimen groups was measured. As shown in Figure 9, the axial residual compressive stress of the Untreated and Nitriding specimens remained relatively uniform, with maximum surface residual compressive stresses of 530 MPa and 671 MPa, respectively. In contrast, the Nitriding-Laser specimen exhibited a decreasing trend in residual compressive stress along the axial direction from the orifice ($L=0,k_{y}\approx 1.0$) inward. At the orifice, the surface residual compressive stress reached 1033 MPa, which is 94.9% and 53.9% higher than those of the Untreated and Nitriding specimens, respectively. The significant increase in surface residual compressive stress in the Nitriding-Laser specimen is a direct result of the LSP process. When the high-energy laser irradiates the ablative coating, it generates a high-pressure plasma shock wave. This wave induces plastic deformation in the surface layer. Upon unloading, the elastic recovery of the subsurface material constrains the plastically deformed surface layer, resulting in the formation of beneficial residual compressive stress. As the peening depth L reached 15.8 mm ($k_{y}\approx 0.5$), the surface residual compressive stress of the Nitriding-Laser specimen decreased to the level of the Nitriding specimen. The gradual decrease in residual compressive stress with increasing depth, as shown in Figure 9, is primarily attributed to the attenuation of the laser shock effect. As the depth increases, $k_{y}$ decreases gradually, and the effect of laser shock peening on promoting plastic deformation of the matrix is weakened accordingly, which ultimately leads to a decrease in residual compressive stress. This relationship confirms that $k_{y}$ is a critical parameter for predicting and controlling the depth-wise effectiveness of the oblique LSP process.

In summary, N-OLSP can significantly enhance surface residual compressive stress on the inner hole. However, the improvement is strongly dependent on the uniformity coefficient, showing that higher uniformity coefficients correspond to more pronounced increases in surface residual compressive stress.

### 3.3. Analysis of Axial Metallographic Structure

**Figure 10:**
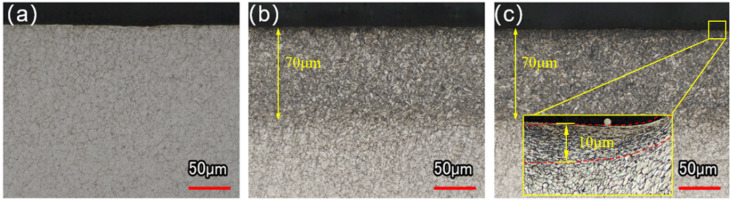
Metallographic structures of specimens: (**a**) Untreated; (**b**) Nitriding; (**c**) Nitriding-Laser.

The metallographic structure of a material significantly influences its mechanical properties, as variations in phase nature, distribution, and grain size affect hardness and stress distribution, which are directly related to wear resistance [30,31]. To investigate the effect of N-OLSP on the inner hole microstructure, the metallographic structures near the orifice (L=0–120 μm) of the three specimen groups were examined. The Untreated specimen primarily consisted of ferrite and dispersed cementite particles, as shown in Figure 10a. Both the Nitriding and the Nitriding-Laser specimens exhibited a nitriding layer approximately 70 μm deep, with a tempered sorbite microstructure, as shown in Figure 10b,c. For the Nitriding-Laser specimen, the intense plastic strain induced by laser shock peening near the surface (peening depth ~10 μm) refined the original coarse grains into smaller grains [32,33]. The grain refinement was most pronounced at the orifice, where the uniformity coefficient was highest, with grain areas reduced to approximately 10 μm^2^ (Figure 10c). As the axial depth increased, the uniformity coefficient gradually decreased, and the grain refinement effect diminished correspondingly.

### 3.4. Analysis of Wear Resistance of Inner Hole

**Figure 11:**
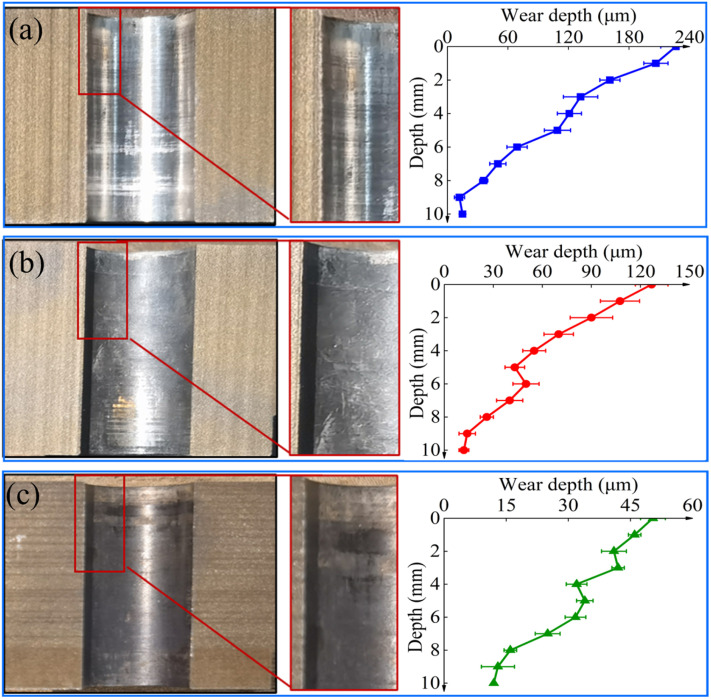
Wear morphology diagrams of the guide hole inner wall: (**a**) Untreated; (**b**) Nitriding; (**c**) Nitriding-Laser.

The wear analysis revealed significant differences in the inner wall condition across the three specimen groups. The Untreated specimen showed pronounced furrows on the inner wall, with the most severe wear occurring at the orifice, where the maximum wear reached 226.33 μm (wear rate 1.81 μm/h), as shown in Figure 11a. The Nitriding specimen displayed surface scratches on the hole wall, with the maximum wear reduced to 126.85 μm (wear rate 0.72 μm/h), as shown in Figure 11b. In contrast, the Nitriding-Laser specimen exhibited only minor friction traces on the inner wall, with a maximum wear of only 50.67 μm (wear rate 0.60 μm/h), representing reductions of 66.85% and 16.67% compared with the Untreated and Nitriding specimens, respectively, as shown in Figure 11c. The above indicates that the wear in all three different specimen groups is most severe near the orifice. Compared with Nitriding specimen, the Nitriding-Laser specimen demonstrates significantly enhanced wear resistance at the orifice [34,35,36].

**Figure 12:**
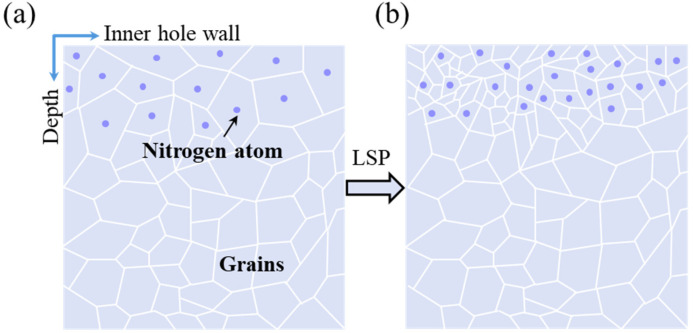
Schematic of microstructural evolution of the guide hole: (**a**) Nitriding; (**b**) Nitriding-Laser.

Figure 12 shows schematic of microstructural evolution of Nitriding and Nitriding-Laser. The Nitriding sample forms a high-hardness nitrided layer (up to 1205 HV_0.1_) on its surface, effectively resisting abrasive attack and reducing wear loss. After composite treatment, the Nitriding-Laser sample undergoes intense plastic deformation in surface and subsurface layers via high-energy density plasma shock waves. This increases dislocation density and refines martensite grains, boosting surface hardness and wear resistance. Additionally, laser shock peening induces residual compressive stress through grain refinement strengthening, which inhibits crack initiation/propagation and surface plastic deformation, reduces wear groove size and spalling risk, and ultimately minimizes wear loss.

## 4. Conclusions

To address the challenge of uneven strengthening of the small hole, this study proposes the strengthening uniformity coefficients k of circumferential and axial direction to optimize the laser oblique impact process. The results show good strengthening effects, yielding the following conclusions:

(1) The axial uniformity coefficient $k_{y}$ at the orifice position is the largest, and the corresponding hardness at the same position is the highest, with a surface hardness up to 1334 HV_0.1_. As the axial depth L increases, the axial uniformity coefficient $k_{y}$ gradually decreases, and the surface hardness of the Nitriding-Laser specimen gradually approaches that of the Nitriding specimen.
(2) N-OLSP significantly enhances the residual compressive stress on the inner hole surface. The improvement is strongly dependent on the uniformity coefficient: higher k values result in greater stress enhancement. The residual compressive stress of the Nitriding-Laser specimen at the orifice increased by up to 94.9% compared with the Untreated specimen.
(3) As the axial depth increases, the uniformity coefficient decreases, leading to a gradual weakening of the grain refinement effect. The grain size at the orifice reaches a minimum of approximately 10 μm^2^, indicating the highest degree of refinement at this location.
(4) N-OLSP markedly improves the wear resistance at the orifice. The maximum wear of the Nitriding-Laser specimen was reduced by 66.85% and 16.67% compared with the Untreated and Nitriding specimens, respectively, effectively suppressing the formation of flared-mouth damage at the orifice.

These findings confirm the uniformity coefficient (k) as a key parameter for the N-OLSP process optimization method to effectively enhance the wear resistance of small holes. The proposed N-OLSP method can be extended to other precision engineering applications involving small holes, though further work is needed to account for radial depth effects and to develop adaptive laser scanning techniques for broader coverage.

While the uniformity coefficient provides a valuable guide for optimizing laser incidence to achieve uniform strengthening in small, deep holes, the present framework has certain limitations. First, the model does not account for the complex thermal accumulation and potential microstructural alterations that could arise from excessively high overlap rates. Second, the enhancement in wear resistance was evaluated under controlled laboratory conditions, and the synergistic effects of corrosive media on the treated surface remain unassessed. These limitations highlight the need for future studies to expand the applicability of the k-based optimization strategy to more complex operational scenarios.

## Figures and Tables

**Table 1:** Chemical composition of H13 steel.

Element	C	Si	Mn	Cr	Mo	V	S	P	Fe
Content (wt%)	0.46	1.10	0.39	4.90	1.21	0.88	0.004	0.01	Balance

**Table 2:** Parameters of OLSP.

Item	Laser Beam Diameter	Energy	Pulse
Value	3 mm	8 J	20 ns

## Data Availability

The original contributions presented in this study are included in the article. Further inquiries can be directed to the corresponding authors.
